# Therapeutics and Pharmacy

**Published:** 1910-01-22

**Authors:** 


### Therapeutics and Pharmacy

### Lead in Syrup of the Iodide of Iron.

The following notes indicate the importance and value of not allowing any unfamiliar peculiarity in a medicine to pass unexplained, or at least to pass without an attempt being made to explain it. A yellow deposit, which ought not to have appeared at all, came down soon after the following prescription was dispensed:—

℞ Syr. ferri iodidi ... ... ... ... } aa ℥ij

Aquam menth. pip. ... ... ... ... }

Misce.

Analyses showed that the precipitate in question consisted of lead iodide. Inquiries showed that the lead had somehow made its way into the syrup of iron iodide in the process of its manufacture, though precisely in what way was difficult to tell. It might have been a result of using lead-enamelled pans, or even of storing the syrup in a vessel that was not clean; but the quantity of lead found suggested that either lead-contaminated iron filings or else crude iodine had been employed in the manufacture of the syrup of iodide of iron. It is important to realise that the syrup itself can appear pure enough, being able to keep a quantity of lead dissolved. It is when the syrup is diluted that the lead comes down. Solutions of iodides have a solvent action on lead, complex salts resulting, and unless the lead be present in fair quantity even a dilution of the liquor with syrup remains quite free of deposit. When water is used decomposition of the double iodide at once follows, and yellow crystals of lead iodide come down as in the above mixture.

### The Black Tongue caused by Bismuth.

Mr. William Duncan has made some interesting investigations into the reasons why some people suffer from blackness of the tongue upon taking bismuth salts in such mixture as the following:—

℞ Glycerini acidi pepsini ... ... ... ʒij

Tincturæ nucis vomicæ ... ... ... ʒij

Liquorem bismuthi ad ... ... ... ℥iv

A black patch about the size of a sixpenny-piece upon the tongue was complained of on the third day after the mixture was begun. The patch steadily increased in size until a strip in the centre of the tongue was quite covered with what appeared to be bismuth sulphide.

It was suggested that tobacco-smoking might in some way be the cause of the deposit, but when the medicine was tried by two ladies who had never smoked their tongues were blackened, even more quickly than were those of smokers.

Normal saliva has been described as containing traces of potassium sulpho-cyanide; but if the blackening is due to sulphur in the saliva it is curious that it should not occur equally in all, and that it should be mainly in the centre of the tongue.

Eructations of sulphuretted hydrogen or other sulphur-containing gas have been suggested; but after special observation upon a number of cases the conclusion arrived at was that the blackening is only met with when the tongue has been coated and foul, the mucus under this condition being apparently rich in sulphur. Blackness of the tongue after taking bismuth, therefore, is equivalent to foulness of the tongue in other circumstances.

